# Automated high-throughput high-resolution X-ray diffraction capabilities at SSRL BL 2-1

**DOI:** 10.1063/4.0001033

**Published:** 2025-10-27

**Authors:** Sikhumbuzo Masina, Monty Cosby, Nicholas Strange, Vivek Thampy, Charles Troxel, Kevin Stone

**Affiliations:** SLAC/SSRL, Menlo Park, CA, USA

## Abstract

High quality powder diffraction data from synchrotron radiation sources remain the cornerstone of structural characterization. They provide insights into the structure of materials; revealing subtle local and average structural details that could otherwise be missed by X-ray laboratory sources. However, high quality diffraction data from many synchrotron radiation sources are not easily accessible. From proposal writing to data collection, the process could take months. Financial and geographical limitations can compound the accessibility problem. The turn-around time for high throughput, mail-in program is significantly shorter than that of an in-person, general user program and is relatively easily accessible.

At the Stanford Synchrotron Radiation Light Source on beamline 2-1, the mail-in program, facilitated by a robotic sample changer can measure up to a 100 *ex situ* samples a day, delivering high throughput and high-resolution data for users [1]. In this work, as a case study, we have worked on high entropy oxides. These are compositionally complex, multifunctional materials that have shown great promise [2]. However, the compositional complexity of these materials makes them challenging to understand at the fundamental level and are prone to phase separation. The phase separation could be subtle and hard to resolve, requiring the high-resolution and high signal-to-noise ratio offered by synchrotron radiation sources. In this work, we have shown how the high-quality data from beamline 2-1, obtained through the mail-in program, could be used to resolve some phase separation observed in high entropy spinel oxides.
